# Prevalence and associated factors of non-medical use of prescription drugs among adolescents in secondary schools in Buea, Cameroon: a cross-sectional study

**DOI:** 10.1186/s12888-023-05120-0

**Published:** 2023-09-25

**Authors:** Cyrille Nkouonlack, Ismaila Ngwayi Shifu, Jonas Guy Basseguin Atchou, Christian Eyoum, Dieudonne Yusinyu Dinayen, Dickson Shey Nsagha, Alfred Kongnyu Njamnshi

**Affiliations:** 1 Department of Internal Medicine and Paediatrics, Faculty of Health Sciences, University of Buea, Molyko, P.O. Box 63, Buea, Cameroon; 2 Brain Research Africa Initiative (BRAIN), PO Box 25625, Yaounde, Cameroon; 3 Faculty of Medicine and Pharmaceutical Sciences, University of Douala, Douala, Cameroon; 4 Psychiatric Department, Military Hospital, Maroua, Cameroon; 5 Department of Public Health and Hygiene, Faculty of Health Sciences, University of Buea, Buea, Cameroon; 6 Neuroscience Lab, Faculty of Medicine and Biomedical Sciences, The University of Yaoundé I, Yaoundé, Cameroon

**Keywords:** Non-medical use, Prescription drugs, Tramadol, Secondary school students, Adolescent

## Abstract

**Background:**

The non-medical use of prescription drugs is a growing public health problem worldwide. Recent trends in Cameroon show that the use of psychoactive substances, among which are prescription drugs by adolescents is becoming a public health issue and is linked to juvenile delinquency and violence in schools. However, there is a paucity of data on the burden of this phenomenon among adolescent secondary school students in the country. The aim of this study was to determine the prevalence and factors associated with the use of non-prescription drugs in secondary schools in Buea, South West region of Cameroon.

**Methods:**

We conducted a cross-sectional study from 1^st^ February 2021 to 30^th^ April 2021. Secondary school students were recruited using a multistage stratified cluster sampling. A modified and standardized version of the World Health Organization student drug-use survey model questionnaire was used. Ethical approval was obtained from the Institutional Review Board of the Faculty of Health Sciences, University of Buea (No. 2021/1273–02/UB/SG/IRB/FHS). The Statistical Package for Social Sciences, IBM SPSS Statistics for Windows, Version 25.0. was used for data analysis. Descriptive statistics were used to describe the sociodemographic characteristics of participants. Univariate and multivariate logistic regression models were used to explore associated factors of non-medical use of prescription drugs.

**Results:**

A total of 570 participants were enrolled for the study, and 510 participants responded giving a response rate of 89.5%. The prevalence of non-medical use of prescription drugs was 15.3%, tramadol being the most used. Motivators for non-medical use of prescription drugs were “to work longer”, “to be courageous”, and “curiosity”. Logistic regression results showed that alcohol consumption [OR 3.68; 95% CI: 2.24–6.06; *p* < 0.001], smoking [OR 6.00; 95% CI: 3.07–11.75; *p* < 0.001] and use of illicit drugs [OR 10.85; 95% CI: 5.48–21.48; *p* < 0.001] were independent factors associated with non-medical use of prescription drugs.

**Conclusion:**

Non-medical use of prescription drugs was prevalent among adolescent secondary school students in Buea, Cameroon. Tramadol is the main drug of prescription involved. Our results can guide policymakers on strategies to screen, prevent and control non-medical use of prescription drugs among secondary school students in Cameroon.

## Background

Non-medical use of prescription drugs (NMUPD), defined as the use of prescription drugs that is not prescribed to a user, or other than in the manner, for reasons, or time period prescribed, is a growing health problem [1, 2]. Young people are more vulnerable to drug use, and the misuse of prescriptions drugs, particularly opioids, benzodiazepines and synthetic stimulants is generally higher among adolescents than among the general population in all regions of the world [3–6].

Globally, about 296 million people 5.8 percent of the global population aged 15 – 64 are estimated to have used drugs in 2021, representing about a 23 per cent increase over the last 10 years [2]. According to data from the United Nations Office on Drugs and Crime (UNODC), there is an increased availability of substances in the drug markets, with NMUPDs ranking second after cannabis [7]. The problematic non-medical use of pharmaceutical opioids, especially tramadol affects many countries worldwide, and is the main driver of non-medical use of prescription drugs in North, West and Central Africa [7–9]. Non-medical use of tramadol is an emerging public health crisis with serious public health consequences in Africa [8–10]. Emerging trends on non-medical use of prescription drugs in Kenya showed that the problem is more prevalent among adolescents and data indicated a lifetime usage of 10.4% among primary school students, with antidepressants, antipsychotics, anticholinergics, opioid analgesics, antihistamines and anaesthetics identified as the main drug classes involved [11]. In Nigeria, a study on substance use among secondary school students revealed that up to 35 per cent of them had taken tramadol, and 18 per cent had taken Refnol in their lifetime for non-medical purposes [12]. Another recent study in Parakou, Benin, reported 13.5% prevalence amongst secondary school students with a lifetime non-medical use of any prescription drugs, mostly among those aged 20 to 24 years [13].

In Cameroon, psychoactive substance use among adolescents and young adults in schools is becoming a public health problem, and is associated with juvenile violence and delinquency [14]. Few studies have been done in tertiary institutions and in street children on recreational drugs and other substance abuse in Cameroon [15–18]. Furthermore, due to conflict and stressors related to internal displacements, there has been an increase in the use of recreational drugs in the country [19]. However, to our knowledge, there has been no investigation on the non-medical use of prescription drugs among adolescents and young people in Cameroon. The aim of the current study was to determine the prevalence and predictors of NMUPD among adolescents in secondary schools in Buea. The data will help in the design of school-based interventions for the prevention and control of non-medical use of prescription drugs among adolescents in secondary schools.

## Methods

### Study design

A cross-sectional study was undertaken in order to determine the prevalence and predictors of non-medical use of prescription drugs in some secondary schools in Buea from 1^st^ February 2021 to 30^th^ April 2021.

### Study area and setting

The secondary schools in Buea have an estimated population of 17,852 students. The city has noticed an increased in its population over the past four years following a socio-political crisis in the anglophone regions of Cameroon that has led to internally displaced persons fleeing from conflicts zones in rural areas to the major city of Buea [19, 20].

### Study population

Adolescent students from seven randomly selected public, mission and lay private secondary schools in the Buea municipality were included.

### Inclusion criteria

All students who accepted to participate in the study and signed a consent/assent form.

### Exclusion criteria

Students aged less than 13 years or greater than 20 years.

### Sample size

The sample size was calculated using the Cochran formula [21]. as shown below;

$$\text{n}= \text{z}^{2}\text{p}\;(1-\text{p})/\text{e}^{2},\ \text{where}\ \text{n}=\text{sample size}$$

*Z* = standard normal variate (1.96) at 95% confidence interval.

*P* = estimate of prevalence of NMUPD was taken as 50% due to paucity of the prevalence study in this population in Cameroon at the time of our study.

e = sampling error that can be tolerated (5%)

$$\mathrm n=(1.96){^2}\;0.5\;(1-0.5)/(0.05){^2}\,=\,384$$

Taking a non-response rate of 10%, 10/100(384) = 39.

Therefore, the least estimated sample size was 423 students, and we recruited an actual sample of 510 students.

Multistage sampling technique was used to obtain study participants. In the first stage, seven secondary schools were conveniently selected out of a total 15 using convenience sampling method in order to include public, lay private and mission schools. Secondly, probability proportionate to size was used to select the number of students from each secondary school. Lastly, simple random sampling (balloting) was used to select students from the individual schools.

### Data collection

Data was collected using a standardized questionnaire, adapted version of the World Health Organization student drug-use survey model questionnaire [22]. This tool has been tested and shown to be reliable and valid among students in a neighbouring country in Nigeria [23]. The questionnaire was pretested among 30 students from secondary schools not selected for the study. Three data collectors were trained on proper data collection steps for a period of four days, and they pretested 10 students each. Following the training, the principal investigator and the 03 data collectors then moved to the various selected schools for data collection. All participants in the study received a participant information sheet. Following clear explanation of the information sheet, the voluntary participants were required to sign an informed consent form or assent form. Schools were visited days before the study and consent/assent forms were presented to administrators who later obtained a verbal consent from the students’ parents/guardians. A self-administered questionnaire was then used to collect the data. After random sampling, students were given about 15 min to fill the questionnaire.

### Ethical approval

Ethical approval was obtained from the Institutional Review Board of the Faculty of Health Sciences, University of Buea (2021/1273–02/UB/SG/IRB/FHS). This was then followed by administrative authorization from the South West regional delegations of public health and of Secondary education respectively. Authorization was also gotten from the various principals of the selected schools. Written consent was obtained from the parents of individual students by the school administration, while in each class, the investigators obtained oral consent from the students in the presence of their class teachers. Participants were informed on the importance of the study and confidentiality and privacy were respected.

### Data management and analysis

Data collection was coded to ensure confidentiality. Data were entered into a computer using the Excel spreadsheet and exported to Statistical package for Social Science (SPSS) version 25 for statistical analysis. Descriptive summary measures were expressed as means ± standard deviation for normally distributed data, or median [interquartile range] for non-normally distributed data and number (percentages) for qualitative variables. Categorical comparisons were performed by Chi-square test. Odds ratios (ORs) and nominal 95% confidence intervals (CIs) were presented. A logistic regression model (multivariate analysis) was used to estimate the association between baseline demographics and predictors for drug use. Statistical significance was set at *p* < 0.05.

## Results

Five hundred and ten out of the five hundred and seventy invited adolescents secondary school students accepted to participate in the study, giving a response rate of 89.5%.

### General characteristics of participants

Most of the participants were form five and upper sixth students, 219 (42.9%) and 201(39.4%) respectively. The majority of the participants were female, 262 (51.4%), and there was an even ratio of 1:1. The mean age (SD) was 17.3 (1.9) years, and the age group most represented was that of adolescent school students between 17 and 20 years (62.4%). The socio-demographic characteristics of the participants are shown on Table 1. Most of the adolescent school students, 325(63.7%) had an estimated monthly allowance of less than 5,000 FCFA, and were mostly living in same homes with their parents or guardians (68.6%).

**Table 1:** Sociodemographic and behavioural characteristics of adolescent secondary school students (*N* = 510)

Variable	Category	Frequency (*N* = 510)	Percentage (%)
Age (Mean ± SD; Range) (years)	17.3±1.9; 17 -20		
Age group(years)	13–16	192	37.6
	17–20	318	62.4
School setting	Public	382	74.8
	Private	128	25.2
Class level	Three	17	3.3
	Four	34	6.7
	Five	219	42.9
	Lower Sixth	39	7.6
	Upper Sixth	201	39.4
Monthly allowance (FCFA)	< 5000	325	63.7
	5–10,000	114	22.4
	10–20,000	34	6.7
	20–30,000	14	2.7
	> 30,000	23	4.4
Number of siblings	1 – 5	397	77.8
	6 – 10	95	18.6
	> 10	8	1.6
Living arrangements	Living with parents	350	68.6
	Living with a Guardian	122	23.9
	Cohabiting with a friend	22	4.3
	Living in a school dormitory	16	3.1
Behavioural Factors	Alcohol use	167	32.7
	Cigarette smoking	42	8.0
	Illicit drug use	42	8.1
Parent`s Marital status	Married	294	57.6
	Single	165	32.4
	Widow	30	5.9
	Divorced	16	3.1
	Cohabiting	5	1.0
Parents`s Employment status	Self-Employed	262	51.4
	Employed	161	31.6
	Unemployed	87	17.1
Parent`s Education status	Primary	34	6.7
	Secondary	272	53.3
	Tertiary	176	34.5
	No formal education	28	5.5

### Prevalence of non-medical use of prescription drugs among adolescent secondary school students

Seventy-eight participants admitted usage of prescription drugs, giving a prevalence of non-medical use of prescription drugs among adolescents in secondary schools in Buea of 15.3%.

**Fig.1:**
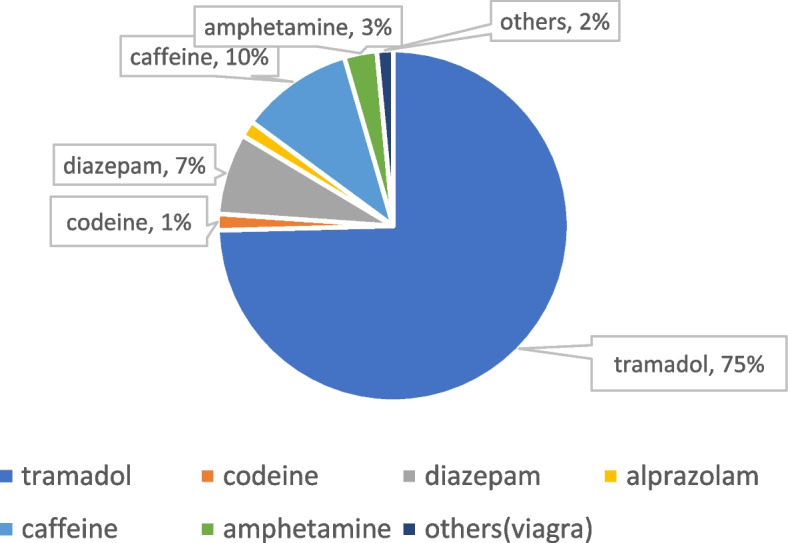
Frequency of common NMUPDs by adolescent secondary school students

The most frequently misused drugs were opioid analgesics, with a prevalence of lifetime usage of 10.58%, followed by stimulants (3.1%), and sleep medication (diazepam) (1.7%). Tramadol was reported to be the commonly used prescription drug (Fig. 1).

Twenty-seven (33.3%) of the students reported a daily usage of prescription drugs for non-medical reasons and 25.9% used them occasionally. More than half of the students [45 (53.6%)] started using the drugs between the ages of 15 and 17 years. These drugs are consumed by students mostly before sexual intercourse [36 (36.7%)], before sporting activities [21 (21.2%)], at parties [19 (19.2%)] and before exams [15 (15.2%)]. These drugs were mostly purchased from roadside vendors (31.7%), local drugstores (23.3%), clubs (18.3%), and approved pharmacies (11.7%).

Non-medical use of prescription drugs were associated with other concurrent substance misuse. Alcohol usage was the most reported substance utilized by the secondary school students 167 (32.8%), followed by tobacco and illicit drug use in 41(8.0%) each. Cannabis was the most common form of illicit substance used by secondary school students, and was reported in 6.07% of the students.

**Fig. 2:**
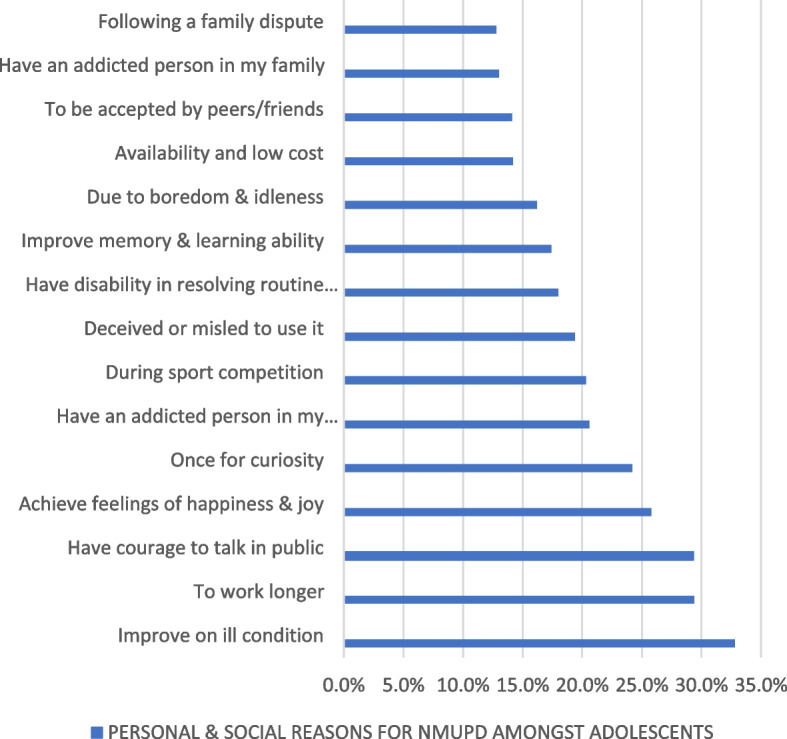
Motivators for NMUPDs among adolescent secondary school students

The factors motivating the non-medical use of prescription drugs were mainly due to personal reasons, or influence from the family, peers or the neighborhood. The motivators for NMUPDs are reported in Fig. 2.

### Predicting factors of NMUPD among secondary school students in Buea

On bivariate analysis, alcohol use (OR 3.68; 95% CI 2.24, 6.06; *P* < 0.000), tobacco smoking (OR 6.00; 95% CI 3.07, 11.75; *P* < 0.000), and use of illicit drugs (OR 10.85; 95% CI 5.48, 21.48; *P* < 0.000), were strongly associated with non-medical use of prescription drugs (Table 2).

**Table 2:** Bivariate analysis of factors associated with NMUPD among Adolescent Secondary School Students

Variable	NMUDP, n (%)		Crude Odds ratio	95% Confidence interval	*P*-value
	**Yes**	**No**			
**Sociodemographic factors**					
Age group (years)					
13–16	24 (12.5)	168 (87.5)	1.00	-	-
17–20	54 (17.0)	264 (83.0)	1.43	0.85–2.40	0.175
Sex					
Male	43 (17.3)	205 (82.7)	1.36	0.84–2.21	0.213
Female	35 (13.4)	227 (86.6)	1.00	-	-
Monthly allowance > 30.000 FCFA	7 (30.4)	16 (69.6)	2.59	1.01–6.63	0.048
Parent`s education status (tertiary level)	32 (18.2)	144 (81.8)	7.33	0.97–55.61	0.054
Live in a school dormitory	4 (25.0)	12 (75.0)	2.00	0.62–6.45	0.246
**Behavioural Factors**					
Use of alcohol	46 (27.5)	121 (72.5)	3.68	2.24–6.06	0.000
Cigarette smoking	19 (46.3)	22 (53.7)	6.00	3.07–11.75	0.000
Use of illicit drugs	24 (58.5)	17 (41.5)	10.85	5.48–21.48	0.000

On multivariable analysis, the use of alcohol (aOR 2.23; 95% CI 1.26, 3.95; *P* = 0.006), and illicit drugs (OR 5.60; 95% CI 2.41, 12.97; *P* < 0.000), were shown to be independently associated with the use of prescription drugs for non-medical purposes among the secondary school students in Buea (Table 3).

**Table 3:** Multivariate analysis of factors associated with NMUPD among adolescent secondary school students

Variable	NMUDP, n (%)	Adjusted Odd Ratio (aOR)	95% CI	*P*-Value
Use of alcohol	46 (27.5)	2.23	1.26–3.95	0.006
Use of illicit drugs	24 (58.5)	5.60	2.41–12.97	0.000

## Discussion

The aim of our study was to determine the prevalence and factors associated with non-medical use of prescription drugs among adolescents in secondary schools in Buea. This is one of the first studies that evaluates the prevalence and correlates of non-medical use of prescription drugs among secondary school students in Cameroon.

### Prevalence of non- medical use of prescription drugs

We found that the prevalence of non-medical use of prescription drugs was 15.3%. Most of the students concerned with NMUPDs were adolescents in the age group between 15 and 17 years (53.6%). Our results indicate that opioid analgesics, specifically tramadol is the most frequently used prescription drug among adolescent secondary school students, followed by stimulants and benzodiazepines. Up to 10.58% of the school students were using opioids, mainly tramadol, followed by stimulants (3.1%), and sleep medication(diazepam) (1.7%).

The use of drugs for non-medical purposes is on the rise among school-age population in Cameroon. Our observation is in keeping with previous findings from tertiary settings in the country, whereby the use of opioid analgesics and other substances such as cannabis and alcohol for recreational purposes is common among youth [15, 18, 19]. Metuge et al. [18] in a study on substance use among students in tertiary institutions in Buea, Cameroon found that the commonest substances used were alcohol (98.4%), tobacco (28.3%), tramadol (7.5%) and 6.8% of cannabis [18]. Their findings are similar to those of Mbanga et al. [15] who also confirmed the association of opioid use with the consumption of other substances as alcohol and tobacco.

Non-medical use of prescription drugs has increased globally over the past decades, and is driven mainly by the increased availability of substances on the drug market [2]. In West, Central, East, and North Africa and the Middle East, although cannabis is the main drug among people with drug use disorders, opioid analgesics remain the main drug involved with non-medical use [24]. Our study findings of tramadol as the main non-prescription drug misused agree with these international trends. Bio-Sya et al. [13] in a cross-sectional study of 384 secondary students within the age group of 10–24 years in Benin, found a lifetime prevalence of non-medical use of tramadol of 9.6%. These findings are similar to the 10.58% of our secondary school students who were using tramadol, although we did not perform urinary toxicological tests for the detection of tramadol and its metabolites as they did. Other authors from Nigeria have reported similar prevalence of non-medical prescription use of tramadol among secondary school students [25–28]. However, our findings are in contradiction with the emerging trends on non-medical use of prescription drugs in Kenya by Kamenderi et al. [11], who reported that the most prevalent prescription drug for non-medical use was diazepam in 35.2% of 68 confirmed samples, followed by flunitrazepam and amitriptyline, while tramadol represented only 1.5% of their samples. We think that the reason for this discrepancy may be that the study in Kenya was a national survey covering up to eight regions, and was not focused only on school students.

### Associated factors of non-medical use of prescription drugs

Most sociodemographic and behavioural factors showed a weak association with non-medical use of prescription drugs. In comparison with other studies [29, 30], we found that the use of alcohol and other illicit drugs such as cannabis were independently associated with the non-medical use of prescription drugs among secondary school students. In Cameroon, alcohol misuse has been shown be accompanied by the use other illicit drugs such as cannabis and tobacco, and is responsible for indiscipline, violence and other harmful effects among adolescents and youth in school settings [31–33]. There is need for further studies to explore the burden of alcohol and other illicit drugs consumption as drivers for the tramadol epidemic that is common in Africa [9, 10], especially among adolescents in secondary school settings. The link between alcohol usage, illicit drug use and other non-medical prescription drugs, especially tramadol is not limited to our setting, it is a global phenomenom, reported in both high-income and low-income countries alike, and most persons who use prescription drugs for non-medical purpose seem to be polysubstance abuses [5, 6, 30, 34, 35].

Like other studies, we found that the desire to achieve emotional feelings of happiness and joy, courage to talk in public, curiosity, peer pressure and the need to improve school performance where the main reasons adolescent school students were engaging in the non-medical use of prescription drugs (Fig. 2). In a systematic review of motivations for the non-medical use of prescription drugs in young adults aged 18 to 25 years, Tess et al. [4], found that most of them are motivated to take prescription drugs such as stimulants, opioids and antidepressants non-medically to experience the cognitive enhancement effects, and to help them study, improve focus, stay alert and to increase energy among other reasons [4]. The few studies done among students in tertiary institutions and other settings in Cameroon have focused only on the use of drugs for recreational purposes [36] and none has explored the motivations behind the non-medical use of prescription drugs, and none of them has focused on the secondary school students despite the violence and delinquency related to their use in these settings [33]. We also found that some of the students had become addicted to the opioids following chronic use for painful medical conditions, for example, recurrent treatment of acute vaso-occlusive crisis pain in sickle cell disease has been associated with subsequent addiction and non-prescription drug usage [37, 38].

### Implication of the findings

This study has made a number of important contributions to the study of non-medical use of prescription drugs among adolescents in secondary schools in Cameroon. Firstly, our study highlights specifically the high prevalence of tramadol misuse in the school setting, which calls for regular screening and monitoring of students. These findings fall in line with the opioid crisis and its impact in Africa [8, 9]. Secondly, our findings have shown some of the factors associated with the non-medical use of prescription drugs in school settings in Cameroon. These findings could help in the design of selective and specific school-based interventions for the prevention and control of the usage non-medical prescription drugs and other illicit drugs in secondary schools. This will contribute to curbing the problem of violence and indiscipline [14], among adolescent secondary school students.

### Strengths and limitations

Our study has the strength of being one of the first studies on the non-medical use of prescription drugs among adolescents, and involves a large sample size from several private and public secondary schools in Cameroon. However, it has some limitations that we must acknowledge. First, the cross-sectional design of our study could not allow us to establish any causal relationship. Secondly, our findings are based on self-reporting by the school students and are therefore subject to recall and information bias. Furthermore, we did not study the impact of conflict and internal displacements of secondary students on non-medical use of prescription drugs in schools. Atabong et al. [19], have shown the use of recreational drugs to be related to conflict in the region and this could be a confounding factor that we did not explore. In spite of these limitations, our findings provide baseline information that could be used in the design of further studies for more evidence that would inform policy in this setting.

## Conclusion

In conclusion, one out of six secondary school students in Buea, South West region of Cameroon, is involved with the non-medical use of prescription drugs. Opioid analgesics, mainly tramadol are the main type of prescription drugs concerned, and alcohol use, tobacco smoking and other illicit drugs are independently associated with non-medical use of prescription drugs. Parents, school authorities and healthcare policymakers need to be aware of the high prevalence of non-medical use of prescription drugs. Our findings can helpful in developing and implementing screening, prevention and targeted school-based interventions among adolescent secondary school students in Cameroon.

## Data Availability

The datasets generated during and/or analysed during the current study are available from the corresponding author on reasonable request.

## Abbreviation

NMUPD: Non-Medical Use of Prescription Drug
